# BAL lymphocytosis as a predictive marker for drug response and long-term outcome in fibrotic ILD: systematic review

**DOI:** 10.1136/bmjresp-2025-004035

**Published:** 2026-06-04

**Authors:** Philipp Suter, Annina E Buechi, Zayne Milena Roa-Diaz, Manuela Funke-Chambour

**Affiliations:** 1 Department of Pneumology, Allergology and Clinical Immunology, Inselspital, University Hospital Bern, Bern, Switzerland; 2 Unity of Pneumology, Division of Internal Medicine and Specialties, Fribourg Hospital and University of Fribourg, Fribourg, Switzerland; 3 Medical Library, University Library of Bern, University of Bern, Bern, Switzerland; 4 Lung Precision Medicine (LPM), Department for Biomedical Research (DBMR), University of Bern, Bern, Switzerland; 5 Division of Pulmonology, Department of Medicine, Lausanne University Hospital (CHUV) and University of Lausanne, Lausanne, Switzerland

**Keywords:** Interstitial Fibrosis, Bronchoscopy

## Abstract

**Background:**

Fibrotic interstitial lung diseases (fILDs) encompass a heterogeneous group of disorders characterised by varying degrees of inflammation and/or fibrosis. The role of bronchoalveolar lavage lymphocytosis (BALL) as a prognostic biomarker and predictor of corticosteroid (CS) response remains uncertain. We investigated whether, in patients with fILD, increased BALL can serve as a marker for CS responsiveness and whether increased BALL may also predict better long-term outcomes following CS therapy compared with patients with lower BALL levels.

**Methods:**

A systematic review was conducted. MEDLINE, Embase, Web of Science and Google Scholar were searched up to 4 August 2025. Eligible studies included mainly observational clinical studies evaluating BALL in fILD, with a focus on short-term CS response (<6 months) as well as long-term survival (>6 months) after treatment, including changes in lung function parameters, radiological response and overall and progression-free survival. Study selection, data extraction and quality assessment were performed independently by two reviewers. Disagreements were resolved by consensus.

**Results:**

30 studies involving a total of 3083 patients were included. Increased BALL was associated with better CS response as reflected by improved lung function and increased survival in fILD subtypes, such as hypersensitivity pneumonitis and non-specific interstitial pneumonia. However, BALL thresholds varied, and study heterogeneity precluded meta-analysis. Most studies showed moderate to high risk of bias.

**Conclusions:**

Increased BALL may predict CS responsiveness and treatment outcome prediction in fILD; however, clinical implementation remains limited due to insufficient evidence, predominantly observational studies, inconsistent BALL thresholds and heterogeneous results across disease entities. Standardisation and prospective validations are needed to better integrate BALL findings into clinical routine.

**PROSPERO registration number:**

CRD420251057631.

**WHAT IS ALREADY KNOWN ON THIS TOPIC**

- Bronchoalveolar lavage lymphocytosis (BALL) is used to assess alveolar inflammation in interstitial lung diseases and has diagnostic relevance in conditions such as hypersensitivity pneumonitis.
- Corticosteroids (CS) are widely used in inflammatory interstitial lung disease (ILD) subtypes; however, their benefit in fibrotic ILD is uncertain.

**WHAT THIS STUDY ADDS**

- Higher baseline BALL is frequently associated with better CS response, reflected by improved lung function, reduced progression or enhanced survival across several fibrotic ILD subtypes, especially fibrotic hypersensitivity pneumonitis and nonspecific interstitial pneumonia.
- Evidence quality is limited and highly heterogeneous, but the overall signal suggests BALL may serve as a potential theragnostic marker; however, further high-quality studies are required before routine clinical implementation can be recommended.

**HOW THIS STUDY MIGHT AFFECT RESEARCH, PRACTICE OR POLICY**

- Findings highlight the need for prospective clinical trials, specifically in fibrotic ILD, to evaluate BALL as a biomarker to guide CS or other immunosuppressive therapy.

## Background

 Fibrotic interstitial lung diseases (fILDs) represent a diverse group of conditions characterised by varying combinations of inflammation and fibrosis within the lung parenchyma.^1^ These conditions present significant diagnostic and therapeutic challenges due to their various clinical presentations and their heterogeneous radiological and pathological presentations. A recent update of the interstitial lung disease (ILD) classification allows better categorisation of clinical, histological and radiological patterns reflecting major advances in understanding pathomechanisms.^2^ Nevertheless, progression prediction and optimising treatment strategies remain challenging.^3 4^

Among the available diagnostic modalities, bronchoalveolar lavage (BAL) provides a distinctive window into pulmonary inflammatory processes without requiring biopsy.^5 6^ Increased BAL lymphocytosis (BALL) is generally considered a marker of alveolar inflammation and may represent a potentially treatable trait in selected fILD phenotypes, with implications for disease characterisation, prognosis and drug response prediction.^7^ However, BALL may coexist with established fibrosis and does therefore not necessarily indicate that inflammatory mechanisms are the dominant drivers of disease behaviour or progression.^8 9^

However, the clinical utility and respective thresholds of BALL in guiding treatment decisions, particularly regarding corticosteroid (CS) therapy in fILD, are based on poor evidence.^10 11^ CS are a cornerstone of treatment for many inflammatory-driven forms of interstitial lung diseases (ILD), yet their role in fILD is more nuanced.^11 12^ While some patients with acute inflammatory presentations respond favourably to CS therapy, patients with idiopathic pulmonary fibrosis (IPF) show adverse outcomes with increased hospitalisation and mortality.^13 14^ If used in fILD other than IPF, CS regimens are not clearly defined, resulting in a wide range of dosing and administration strategies.^10^ Moreover, systemic CS are associated with significant side effects, especially when given over prolonged periods of time.^15^ Defining biomarkers that can reliably predict CS responsiveness would be critical to tailoring therapy and minimising unnecessary exposure to potentially harmful treatments. BALL has been proposed as such a biomarker, while better treatment responses in specific subgroups of ILD have been reported if increased BALL levels are present.^9 10 16^

Despite the promise of BALL as a tool for risk stratification and treatment guidance, its role in clinical practice is far from established. The interpretation of BALL is complicated by several factors, including the inherent heterogeneity of ILDs, variability in BAL procedures and a lack of standardised thresholds to define alveolar lymphocytosis.^6^

This systematic review seeks to address the critical gap in understanding the role of BALL in fILDs, with the aim of synthesising the evidence around its utility as a biomarker guiding responsiveness to CS therapy in patients with fILD focusing on clinical, functional and radiologic outcomes, as well as its prognostic value for long-term outcomes.

## Methods

### Search strategy and data collection

A systematic literature search was conducted across MEDLINE (via Ovid), Embase (Elsevier) and the Web of Science Core Collection (Clarivate) to identify relevant studies. Google Scholar was used to complement the primary database searches and was limited to the first 200 results ranked by relevance, in line with established methodological recommendations.^17^ The search was performed from inception through 4 August 2025. No language restrictions were applied. The detailed search strategy is provided in the online supplemental file 1. The study report adhered to the Preferred Reporting Items for Systematic Review and Meta-Analysis statement standards (online supplemental file 2). The study protocol was registered in the PROSPERO international prospective register of systematic reviews under CRD420251057631. We were unable to perform a meta-analysis with reliable pooled estimates due to substantial heterogeneity among the studies.^18^,^20^ We conducted qualitative synthesis instead.

Two independent reviewers screened all titles and abstracts for potentially relevant articles and extracted data using Covidence (Covidence, Veritas Health Innovation, Melbourne, Australia). Articles not published in English, French or German were translated using DeepL Translator (DeepL SE, Cologne, Germany). Furthermore, the investigators manually reviewed the reference lists of the included articles to identify additional relevant studies. Disagreements between the two reviewers were resolved by discussion.

### Study selection and data extraction

We included studies evaluating the short-term response after CS and long-term outcome in adults with a diagnosis of fILD and BALL, focusing on lung function, survival or mortality rate, respectively, or radiologic pattern evolution. Short-term response was defined as outcomes assessed during hospitalisation or within the first 6 months, whereas long-term outcomes were defined as those assessed beyond 6 months.

Optimal BALL thresholds were extracted as reported in the original studies. However, threshold determination was based on study-specific endpoints and heterogeneous analytical strategies. Inclusion and exclusion criteria and data extraction methods were detailed in the online supplemental file 3. Heterogeneity was assessed narratively following the Synthesis Without Meta-analysis (SWiM) guidelines by comparing study characteristics such as ILD subtype, CS regimen, BALL thresholds and study design.^21^ No statistical measures of heterogeneity were calculated, as data were too heterogeneous for quantitative synthesis. The results were reported in line with the SWiM guidelines (online supplemental file 4).^21^ Harvest plots were generated to display the direction of effects alongside methodological robustness.^21 22^

### Risk of bias assessment

The two reviewers independently assessed the risk of bias of the included studies using the Newcastle-Ottawa Quality Scale (NOS), which assesses studies across three domains: selection of participants, comparability between study groups and ascertainment of exposure for case-control studies or outcome for cohort studies, respectively.^23^ Studies achieving eight points or more on the NOS were considered low risk of bias; seven to six points moderate risk of bias and five points or less high risk of bias. This categorisation reflects a pragmatic approach consistent with prior reviews, rather than the application of formally validated NOS cut-off thresholds. It enables transparent stratification and narrative interpretation of study quality.^23 24^

## Results

**Figure 1:**
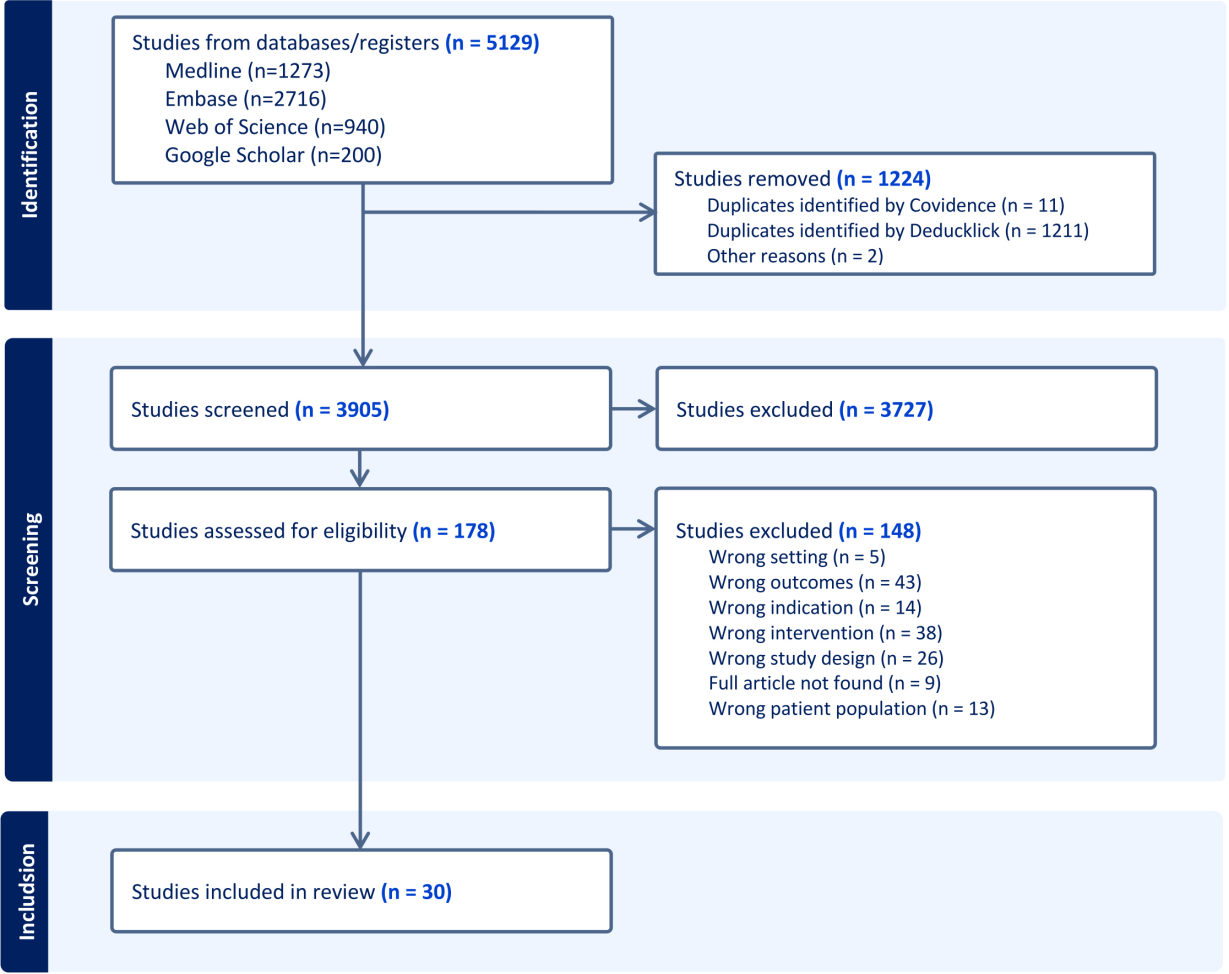
PRISMA flow diagram for study inclusion. PRISMA, Preferred Reporting Items for Systematic Reviews and Meta-Analysis.

The initial literature search yielded 5129 studies. Of these, 1224 duplicated articles were excluded, leaving 3905 articles for screening. Figure 1 outlines our research methodology and selection process. After title and abstract screening, we retained 178 articles for full text screening. We retained 30 articles for our final review (table 1). Out of these, 18 studies were retrospective, while 12 studies were prospective cohort studies. Included manuscripts were published between 1980 and 2024.^25^,^54^ The studies collectively analysed data from a total of 3083 patients, of whom 2556 underwent BAL. Among these, 1756 patients received immunosuppression (IS) therapy and 1198 oral CS, while the remaining patients received various combined IS regimens. IS treatment combinations included CS added to azathioprine, cyclophosphamide, cyclosporine, penicillamine, mycophenolate mofetil, methotrexate or other non-specified IS drugs.^26^,^2834^ In most included studies, BAL was performed prior to initiating CS or IS therapy, and only a minority of patients were receiving IS at the time of BAL (table 1, online supplemental file 5).

**Table 1:** Description of included studies

Author	Study type	Disease	Number of patients (n)/patients (n) with BAL	Smoking status (n=active/ former/never)	IS/CS before BAL or intervention (n)	CS/other IS	Follow-up time	Risk of bias
Bacha *et al*^30^	Prospective ChS	Sarcoidosis	40/40	13/0/27	No	NR/NR	12±9 months	High (S3 C0 O2)
Behr *et al*^31^	Prospective ChS	SSc	79/79	3/4/72	No (72), not for at least 3 months before IS (7)	Prednisone/NR	56.8±3.1 weeks	High (S3 C1 O2)
Cho *et al*^27^	Retrospective ChS	NSIP	204/159	18/29/157	NR	NR/azathioprine, cyclophosphamide	70.9±10.8 months	High (S2 C0 O2)
Cho *et al*^52^	Retrospective ChS	IPF/SARD-ILD	31/31	14/0/17	No	NR/NR	3–18 months	High (S2 C0 O2)
De Sadeleer *et al*^32^	Retrospective ChS	fHP	91/91	NR	No	NR/NR	Lung function at 12 months or treatment interruption, survival 130 months	Moderate (S3 C1 O3)
Drent *et al*^33^	Retrospective ChS	Sarcoidosis	26/26	0/0/26	No	NR/NR	9–34 months	High (S2 C0 O3)
Giacomelli *et al*^34^	Prospective ChS	SSc	23/23	0/0/23	No	Prednisone/ cyclophosphamide	6 months	High (S2 C0 O2)
Goh *et al*^35^	Retrospective ChS	SSc	141/141	0/45/96	Yes 36, No 105	Prednisone/ azathioprine, mycophenolate mofetil, cyclophosphamide	10 years	High (S2 C0 O2)
Greene *et al*^36^	Prospective ChS	SARD-ILD	36/36	12/24/0	No	Prednisone/ cyclophosphamide	Lung function 2 months, clinical evaluation 18 months	High (S3 C0 O2)
Haslam *et al*^26^	Retrospective ChS	IPF, SARD-ILD	66	24/12/15	Yes 10, No 26, NR 30	Prednisone/NR	12 months	Moderate (S3 C1 O3)
Haslam *et al*^25^	Prospective ChS	IPF, asbestosis	21/21	7/8/6	Yes 3, No 18	Prednisone/NR	12 months	Moderate (S3 C0 O3)
He *et al*^29^	Retrospective ChS	Dermatomyositis-ILD	113/113	18/0/95	Yes 83, No 30	Methylprednisolone/NR	22±14.9 months	Moderate (S3 C0 O3)
Kang *et al*^37^	Prospective ChS	IPF	20/20	20/0/0	NR	Prednisone/ cyclophosphamide	6 months	Moderate (S3 C0 O3)
Karpel *et al*^38^	Prospective ChS	IPF	18/18	0/3/15	No	Prednisone/NR	2±0.5 months	Moderate (S4 C1 O2)
Kase *et al*^53^	Retrospective Chs	SSc	68/68	3/22/43	Yes 20, No 48	Prednisone/ cyclophosphamide, methotrexate	88±30 months	Moderate (S3 C1 O3)
Kono *et al*^39^	Retrospective ChS	AE-ILD	71/71	5/44/21	Yes 10, No 61	Methylprednisolone/NR	3 and 12 months	Moderate (S3 C0 O3)
Kurasawa *et al*^40^	Prospective ChS	Dermatomyositis and polymyositis-ILD	22/22	NR	No	Prednisone/NR	12–48 months	High (S2 C0 O2)
Kyung *et al*^41^	Retrospective ChS	IPF	36/36	9/12/11*	NR	NR/NR	3 years	Moderate (S3 C1 O3)
Lewandowska *et al*^28^	Retrospective ChS	HP	93/78	78†/0/15	NR	Prednisone/ azathioprine	6–12 and 200 months	Moderate (S3 C1 O2)
Li *et al*^42^	Retrospective ChS	IPF	126/126	75/0/51	NR	NR/NR	29.6 months	Low (S3 C2 O3)
Matsuo *et al*^43^	Retrospective ChS	IIP	35/29	NR	No	Prednisone/NR	NR	High (S2 C0 O2)
Newman *et al*^44^	Prospective ChS	Beryllium disease	110/110	48/39/23	Yes 10, No 100	Prednisone/NR	NR	High (S2 C0 O2)
Novoa-Bolivar *et al*^54^	Retrospective ChS	HP, sarcoidosis, COP, LIP, RB-ILD, DIP, NSIP, pneumoconiosis, PLCH, eosinophilic ILD, unclassifiable ILD, AIP	1074/371	NR	NR	NR/rituximab, azathioprine, mycophenolate mofetil, cyclophosphamide, tacrolimus	NR	Moderate (S3 C1 O3)
Onishi *et al*^45^	Retrospective ChS	OP	75/75	6/32/36*	Yes 75	Prednisone/NR	NR	Moderate (S3 C0 O3)
Rudd *et al*^46^	Prospective ChS	IPF	120/36	NR	No	Prednisone/ cyclophosphamide, azathioprine, penicillamine	38 months	Moderate (S2 C0 O2)
Sharma *et al*^47^	Retrospective ChS	Chronic silicosis	51/38	18/0/33	No	Prednisone/NR	1.5, 3, 6 months	High (S2 C0 O2)
Takei *et al*^48^	Retrospective ChS	AE chronic fILD	37/28	4/23/9*	Yes 6, No 31	Methylprednisolone/ cyclophosphamide, cyclosporine	6.9±4.4 months	High (S3 C0 O2)
Turner-Warwick *et al*^49^	Prospective ChS	IPF and SARD-ILD	32/32	26/0/6	Yes 6, No 26	Prednisone/ cyclophosphamide	4±1.5 years	High (S2 C1 O2)
Watters *et al*^50^	Prospective ChS	IPF	26/26	12/0/14	No	Prednisone/NR	6 and 12 months	Moderate (S3 C1 O2)
Yamagata *et al*^51^	Retrospective ChS	NSIP, PPFE and unclassifiable IIP	186/186	100/0/86	No	NR/NR	4.4±2.3 years	Moderate (S3 C1 O3)

* Incomplete data.

† Ever-smoker.

AE-ILD, acute exacerbation of interstitial lung disease; AIP, acute interstitial pneumonia; BALL, bronchoalveolar lavage lymphocytosis; C, comparability between groups; ChS, cohort study; COP, cryptogenic organising pneumonia; CS, corticosteroids; DIP, desquamative interstitial pneumonia; DLCO, Diffusing Capacity of the Lungs for Carbon Monoxide; DM, dermatomyositis; fHP, fibrotic hypersensitivity pneumonia; FVC, forced vital capacity; HP, hypersensitivity pneumonitis; HRCT, high-resolution computer tomography; IIP, idiopathic interstitial pneumonia; IPF, idiopathic pulmonary fibrosis; LIP, lymphocytic interstitial pneumonia; N, number of patients; (n), number of patients; NR, not reported; NS, non-significative; NSIP, fibrotic non-specific interstitial pneumonia; O, outcome; OP, organising pneumonia; PFT, pulmonary function test; PLCH, pulmonary Langerhans cell histiocytosis; PM, polymyositis; PPF, progressive pulmonary fibrosis; PPFE, pleuroparenchymal fibroelastosis; RB-ILD, respiratory bronchiolitis-ILD; S, selection of participants; SARD-ILD, systemic autoimmune rheumatic diseases-associated interstitial lung disease; SSc, systemic sclerosis; VC, vital capacity.

The number of patients (n) included in each study ranged from 18 to 1074.^27 54^ The underlying diseases included IPF, systemic autoimmune rheumatic disease-associated interstitial lung disease (SARD-ILD), hypersensitivity pneumonitis (HP), fibrotic idiopathic non-specific interstitial pneumonia (NSIP), sarcoidosis and other fILDs.

Regarding the risk of bias of the included cohort studies, 1 had a low risk of bias, 15 had a moderate risk of bias and 14 had a high risk of bias.

### BAL lymphocytosis as a predictor of corticosteroid response

We identified 22 studies investigating BALL as a predictive biomarker of a CS response in patients with fILD, 9 were prospective and 13 retrospective cohort studies. A total of 2489 patients, of whom 1527 received IS treatment, were included (table 2).^25^,^2831^ Of the selected studies, 1 was rated as having a low risk of bias, 12 as moderate, and 9 as high.

**Table 2:** BAL lymphocytosis as a predictor of corticosteroid responsiveness

Article	Intervention (N)	Type of control (N)	Primary outcome	Time to longest follow-up (months)	Risk of bias	Summary of results
Behr *et al*^31^	CS (38)	NI (41)	PFT, DLCOc, PaO2	14.2±0.8	High	BALL predicted better response: **VC −11.4% pred versus +2.0% pred DLCO −11.2% versus +0.8% exercise PaO_²_ −4.4 versus +1.2 mm Hg, composite index −26.9 versus +4.0**
Cho *et al*^27^	197 (52 CS, 94 CS and azathioprine, 20 azathioprine, 3 cyclo-phosphamide, 1 others)	NT (7)	Progression (Def of PPF)	3–18	High	Factors associated with lower progression/relapse risk: **BALL (aHR 0.59) and CS+AZA (aHR 0.56)**
Cho *et al*^52^	CS (21)	NT (10)	Physiologic, radiologic and pathologic severity	70.9±10.8	High	Initial BALL higher in responders **26.6%±10.7 versus 11.8%±8.8**
De Sadeleer *et al*^32^	CS (67)	NT (24)	Survival, CT progression, PFT	~130*	Moderate	**FVC improved after CS in BALL >20% (+5.66%)** but not in BALL ≤20% DLCO unchanged **Low BALL associated with worse OS (HR 2.66)**
Drent *et al*^33^	CS (8)	NT (28)	HRCT, PFT, 67Galium uptake	9–34	High	BALL in spontaneous recovery 40.4%±4.98 response to CS 19.7%±4.16 deterioration 40.2%±12.92
Greene *et al*^36^	CS or cyclo-phosphamide	No control	Clinical outcome/mortality	18*	High	Patients with BALL on CS had better prognosis
Haslam *et al*^26^	CS and other IS treatment	15 smoking healthy controls	Clinical outcome	12	Moderate	**BALL >11% related to improvement under CS**
Haslam *et al*^25^	CS	No control	Clinical outcome	12	Moderate	BALL >5% predicted response to CS/IS
Kang *et al*^37^	CS (14), CS+cyclo-phosphamide (6)	No control	Clinical outcome (dyspnoea, PFT, HRCT)	6	Moderate	BALL higher in IS responders **23.8%±16.3 versus 7.8%±3.6**
Karpel *et al*^38^	CS (10)	Healthy control (10)	DLCO	2±0.5	Moderate	CS improved DLCO in patients with BALL
Kono *et al*^39^	CS	No control	Clinical outcome	3, ~12*	Moderate	BALL >25% predicted favourable OS **univariate analysis 0.958 (0.932–0.981) multivariate analysis 0.968 (0.936–0.994**)
Kurasawa *et al*^40^	CS	No control	Clinical outcome	12–48*	High	No difference between BAL >15% and <15%
Lewandowska *et al*^28^	CS (76), CS+azathioprine (17)	No control	Clinical outcome (PFT, chest X-ray)	~200*	Moderate	BALL >54% predicted favourable outcome: **AUC 0.71 (95% CI 0.598 to 0.71);** **OR 8.3 (95% CI 2.24 to 3.79)**
Li *et al*^42^	82 CS, 12 CS+cytotoxic drugs	NT (32)	Mortality	29.6	Low	Mortality risk with a BALL: **0.96 (SD 0.177)**
Matsuo *et al*^43^	CS (16)	NT (19)	Mortality	NR	High	BALL >15%: CS group 5-year mortality 52.8% versus 77.4% responders 12.6% versus non-responders 22.8%
Novoa-Bolivar *et al*^54^	CS (234), IS (132)	NT (234)	Survival	NR	Moderate	OS without IS: **BALL >7%, neutrophils <5% (group 1): 11.5 years ± 0.98** **BALL <7% or neutrophils <5% (group 2): 5.8±1.3 years** **Others (group 3): 7.7 years±0.76** OS with CS: **Group 1: 10.0±2.5** **Group 2: 1.7±0.4** **Group 3: 4.4±2.1** OS with IS **Group 1: 9.9±2.5** **Group 2: 2.5±0.7** **Group 3: 6.4±1.3**
Onishi *et al*^45^	CS	No control	Relapse	NR	Moderate	BALL 51.8±19 versus 59.3±20.4 in CS responders
Rudd *et al*^46^	CS (79), 12 other drugs (cyclo-phosphamide, azathioprine, penicillamide)	NT (29)	PFT, BAL results, survival	~38*	Moderate	**Higher BALL in responders versus non-responders, only with CS**
Takei *et al*^48^	CS (13), CS+cyclo-phosphamide (2), CS+cyclosporine (10), CS+cyclosporine + cyclo-phosphamide (11)	No control	Mortality	3, 6.9±4.4	High	BALL >15% with lower 90-day mortality **HR 0.125; 0.0247–0.589**
Turner-Warwick *et al*^49^	CS (15), CS+cyclo-phosphamide (11)	NT (6)	Clinical outcome	48±18	High	Better improvement with BALL >11% versus <11%
Watters *et al*^50^	CS (22)	NT (4)	Combined score (PFT, Rx, dyspnoea, SpO2)	12	Moderate	**BALL >13% associated with clinical improvement and higher clinical scores** **55.5 versus 27.4**
Yamagata *et al*^51^	CS (39), CS+other IS (84)	NT (63)	Overall survival, PFT	52.8±27.6	Moderate	BALL >15% versus no BALL: better prognosis **HR 0.26, 95% CI 0.11 to 0.63** **Higher FVC slope** **Longer OS** Multivariate: **BALL >15% prognostic for OS HR 0.31, 95% CI 0.13 to 0.75.** BALL no prognostic value for OS without IS

* Longest evaluation point in a survival or prognosis analysis, significant results as reported (p<0.05 or 0.01) marked in** bold**.

aHR, adjusted hazard ratio; BAL, bronchoalveolar lavage; BALL, bronchoalveolar lavage lymphocytosis; CS, corticosteroids; DLCO, diffusing capacity of the lungs for carbon monoxide; FVC, forced vital capacity; HRCT, high-resolution computer tomography; IS, immunosuppression; NI, no intervention; NS, non-significative; NT, no treatment; OP, organising pneumonia; OS, overall survival; PaO2, partial pressure of oxygen; PFT, pulmonary function test; PM, polymyositis; PPF, progressive pulmonary fibrosis; VC, vital capacity.

**Figure 2:**
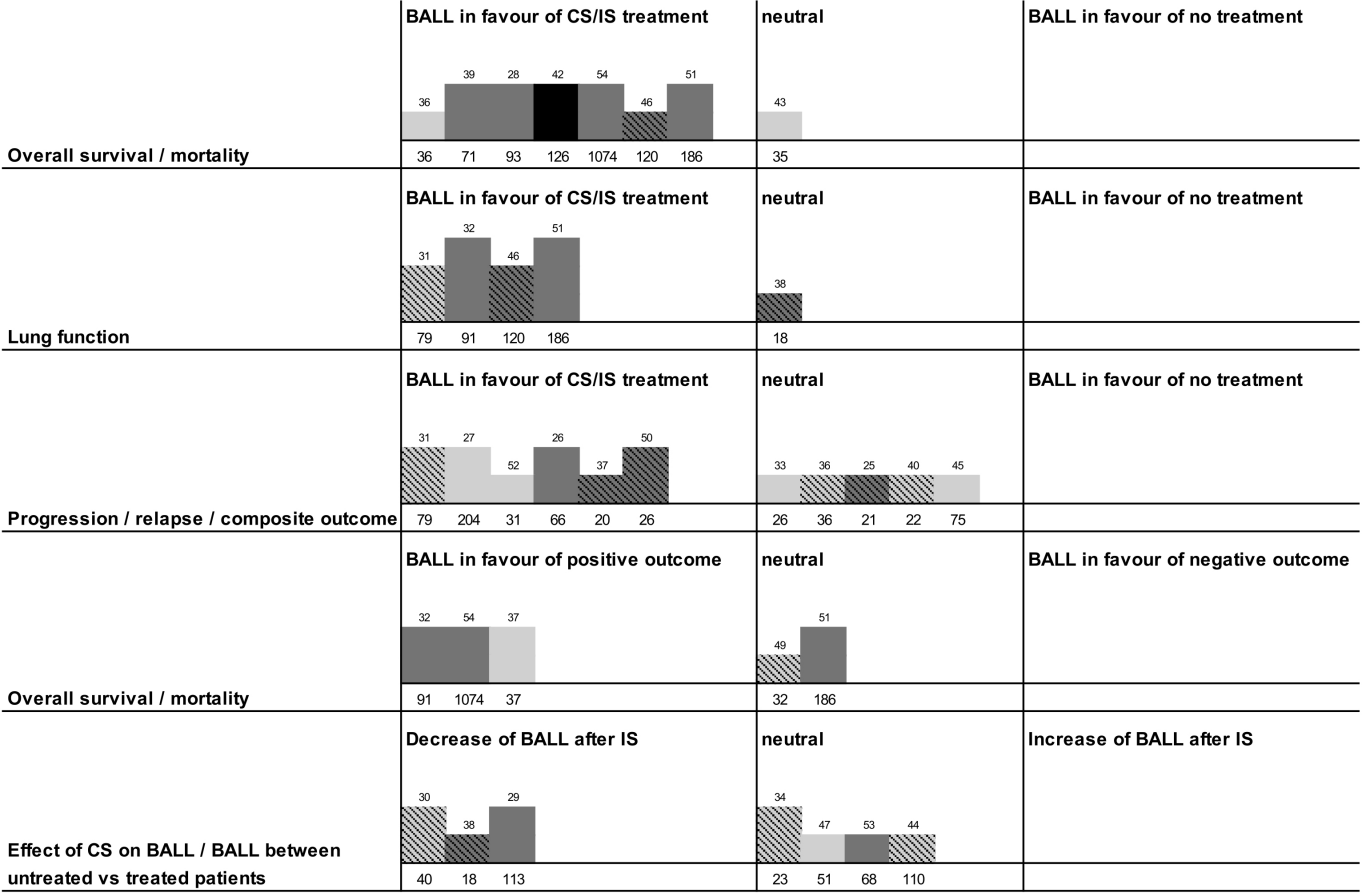
Harvest plot summarising the direction and strength of evidence. Each bar indicates a study, referenced above the bar. Bar height represents the methodological suitability of each study in answering the research question. Bar colour reflects risk of bias (black=low, dark grey=moderate, light grey=high). Striped bars indicate prospective studies, solid bars indicate retrospective studies. Numbers below the bars indicate the total study sample size (number of patients) of the respective study. Numbers above the bars indicate the reference. BALL, bronchoalveolar lavage lymphocytosis; CS, corticosteroids; IS, immunosuppression.

The studies frequently focused on composite clinical outcomes, including pulmonary function tests, exercise-induced partial oxygen pressure, radiologic severity and mortality.^31 32 36 46 52 54^ The direction and methodological strength of the evidence are displayed in the harvest plots (figure 2).

In studies focusing on compound outcomes, higher BALL has been associated with a more favourable outcome compared with lower BALL levels. This finding has been observed in granulomatous diseases such as fibrotic HP but also in SARD-ILD and IPF.^25 26 28 32 37 38 40 46 49 50^ This contrasts with the findings of Greene *et al*, who identified lower BALL in patients with a SARD-ILD as a more favourable prognostic factor than higher BALL.^36^ Notably, this study had a high risk of bias.

De Sadeleer *et al*^32^ reported, in a retrospective study with moderate risk of bias, a positive effect of CS on forced vital capacity (FVC), with an increase of +5.66% (p=0.004) in patients with fibrotic HP and with a BALL >20%. This improvement was observed early after the introduction of CS. However, follow-up revealed that the FVC decline in the treated group progressed along a trajectory similar to that observed prior to treatment. No effect on corrected diffusing capacity of the lung for carbon monoxide was observed.^32^

Cho *et al* evaluated, in a retrospective study with a high risk of bias, patients with fibrotic idiopathic NSIP and SARD-ILD with fibrotic NSIP pattern.^27^ Patients with increased BALL under IS treatment had a lower risk of clinical and functional disease progression compared with those who did not receive IS.^27^ In a retrospective study with a moderate risk of bias, Yamagata *et al* reported that among patients with idiopathic NSIP, idiopathic pleuroparenchymal fibroelastosis (PPFE) and unclassifiable idiopathic interstitial pneumonia (IIP), those with initially increased BALL who received IS had a better prognosis, reflected by higher FVC slopes and longer overall survival. In contrast, IS did not show similar beneficial effects in patients with low BALL counts.^51^

In the study by Behr *et al*,^31^ patients with systemic sclerosis (SSc) who exhibited increased inflammatory cells in their BAL (defined as BALL >15% or neutrophils >5%) experienced significantly worse FVC and DLCOc declines, as well as a higher incidence of exercise-induced hypoxaemia, compared with those with lower BAL inflammatory activity. Notably, patients with increased inflammatory cells demonstrated stabilisation or improved lung function following IS treatment.^31^

Studies involving patients with radiological fibrosis have demonstrated an improvement of various clinical outcomes after CS initiation when baseline BALL counts were increased.^25 28 37 38 40 49 50^ Although one study included radiological response as part of a composite outcome, no study assessed evolution of CT imaging as an independent endpoint to assess CS response.^37^

Two studies analysed the risk of treatment failure or risk of disease relapse related to baseline BALL counts.^27 45^ Cho *et al*^27^ showed that patients with histologically confirmed NSIP, including both idiopathic NSIP and connective tissue disease-associated fILD, who had higher initial BALL (>15%) exhibited a significantly lower risk of disease progression or relapse compared with those with lower initial BALL counts. Many of these patients received CS-based therapies, frequently in combination with azathioprine.^27^ However, this retrospective study was judged to be at high risk of bias.

Overall, patients with increased BALL counts receiving CS treatment demonstrated improved survival compared with those with lower BALL counts and CS treatment.^42 46 51 54^ In a large retrospective study with a moderate risk of bias, Novoa-Bolivar *et al* reported that patients with fILD with higher baseline BALL counts had significantly improved overall survival with CS or IS treatment, whereas low BALL or increased BAL neutrophils was associated with worse outcomes.^54^ In contrast, Matsuo *et al* reported no prognostic impact of BALL (>15%) on 5-year survival in a cohort of patients with IIP, the majority of whom were treated with CS.^43^

In the context of acute exacerbations of ILD (AE-ILD), historically patients were often treated with high-dose pulse CS. In this context, increased BALL was associated with improved short-term survival.^39 48^ In a retrospective cohort study of moderate risk of bias including patients with IPF, SARD-ILD, fibrotic HP or other non-IPF fibrotic lung diseases, Kono *et al* identified increased BALL as an independent favourable prognostic factor.^39^ Takei *et al* similarly reported improved 90-day survival in patients treated with IS if BALL counts were increased at baseline compared with patients with low initial BALL during acute exacerbation.^48^

### Thresholds of bronchoalveolar lavage lymphocytosis: variability across studies

**Figure 3:**
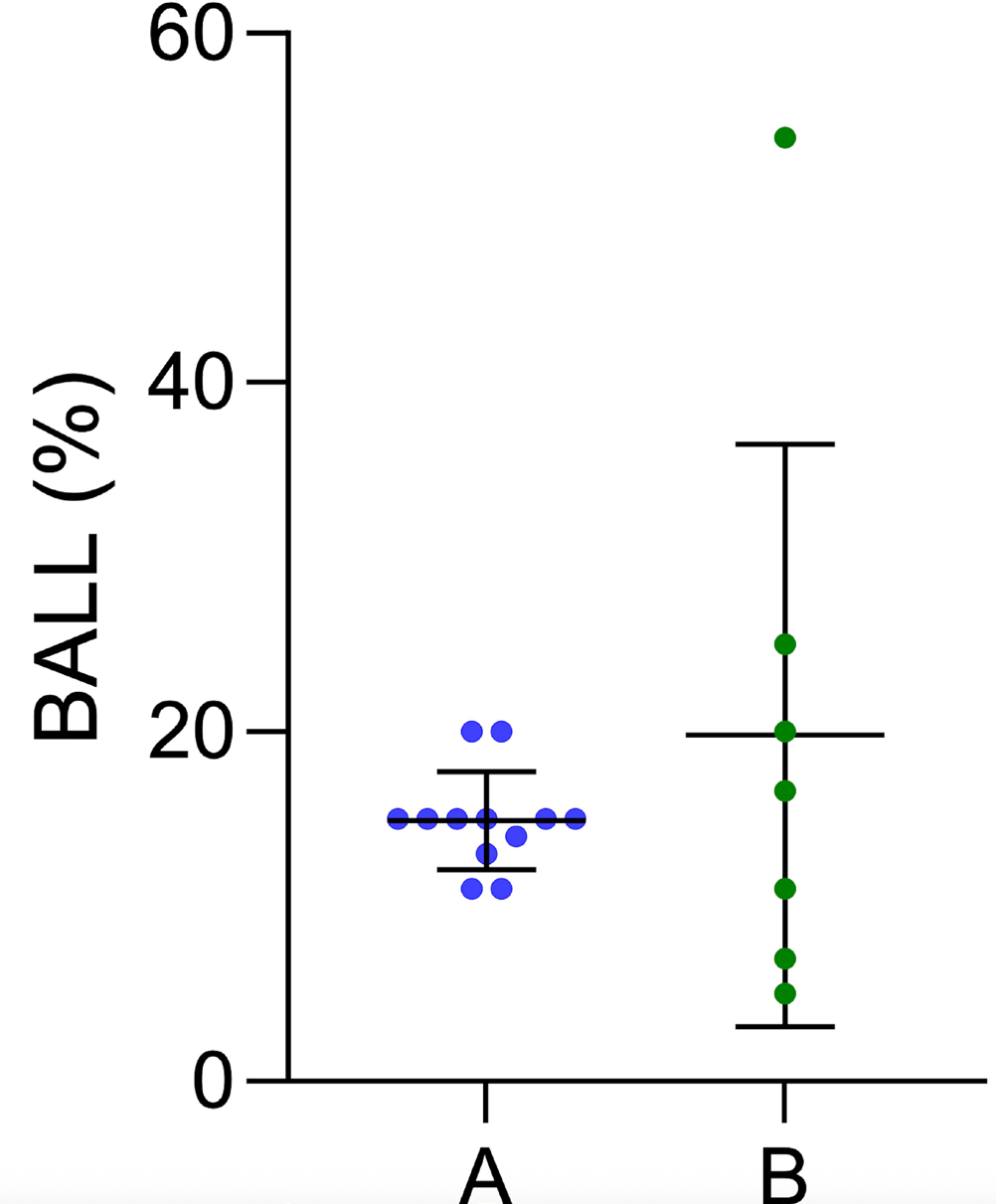
Scatter plots of BALL threshold, with mean±SD. BALL thresholds were extracted as reported in the original studies, which used study-specific outcomes and heterogeneous analytical methods. (**A**) Arbitrary selected BALL thresholds (11–20%). (**B**) BALL thresholds associated with best prognostic yield (5–54%). BALL, bronchoalveolar lavage lymphocytosis.

We identified 12 studies evaluating CS treatment in ILD that used a predefined threshold for BALL, which ranged from 11% to 20%.^2627 31 32 35 40 43 46 48^,^51^ Seven studies derived population-specific optimal BALL thresholds concerning survival, disease progression or lung functional changes (figure 3, online supplemental file 6).^25 26 28 32 39 51 54^

Most studies were rated at moderate risk of bias and collectively included 2310 participants, of whom 1331 received IS treatment. Notably, there was considerable variability in the reported thresholds for BALL across studies. De Sadeleer *et al* showed that a 20% BALL threshold yielded the best prognostic discrimination compared with higher values, whereas Lewandowska *et al* identified a much higher optimal threshold (>54%) to differentiate fibrotic HP from non-fibrotic HP.^28 32^ In the study by Novoa-Bolivar *et al*, analysing a broad spectrum of ILDs, the authors reported an optimal BALL threshold of >7% for predicting overall survival, but a specific cut-off for the fILD subgroup was not determined.^54^

Kono *et al* focusing on patients with AE-ILD identified a 25% BALL threshold to predict response to CS therapy.^39^ In contrast, studies involving patients with IPF or mixed ILD phenotypes (iNSIP, PPFE, asbestosis, unclassified IIP or SARD-ILD) generally reported lower BALL threshold ranging from 5% to 16.6%.^25 26 51 54^

### Effect of corticosteroid therapy on bronchoalveolar lavage lymphocyte levels

Seven studies with a total of 423 patients, including 120 controls, have investigated changes in BALL following the initiation of CS therapy. Overall, these studies reported a general reduction in BALL counts after treatment, although the magnitude of the response varied significantly depending on the underlying disease (figure 2, online supplemental file 7).^29 30 34 38 44 47 53^ Importantly, none of the predominantly retrospective cohort studies evaluated BALL evolution as a primary outcome, and most were rated at high risk of bias.

In the study by Bacha *et al* which included some patients with fibrotic sarcoidosis, a notable decrease in BALL was observed in patients treated with CS, particularly in those with high baseline BALL.^30^ In contrast, Sharma *et al* investigated chronic silicosis and reported only a non-significant trend towards BALL reduction.^47^ However, both studies demonstrated a high risk of bias.

Other studies compared BALL levels between CS-naive patients and those previously treated with CS. A retrospective study with moderate risk of bias, He *et al* found that patients with acute exacerbations of dermatomyositis-ILD who had previously received CS therapy exhibited lower BALL levels.^29^ Conversely, in diseases such as berylliosis or SSc, no significant difference in BALL was observed between treated and untreated patients.^44 53^

Similarly, Giacomelli *et al* reported no significant effect of CS on BALL; however, it is worth noting that patients with SSc in this cohort had lower baseline BALL levels compared with the above-mentioned studies.^34^

## Discussion

To our knowledge, this review is the first to systematically evaluate the role of BALL in predicting CS treatment response and its value as a marker for short- and long-term outcomes following CS therapy in patients with fILD. Our analysis suggests that increased BALL might serve as a predictive marker for a more favourable CS response, with several studies demonstrating improved clinical outcomes, including better survival, slower pulmonary function decline and reduced disease progression in patients with higher BALL levels. However, some conflicting evidence exists, and reported outcomes vary considerably depending on disease subtype and study quality.

IPF holds a distinct position due to the findings of the PANTHER-IPF study, which reported increased mortality with combination therapy of prednisone, azathioprine and N-acetylcysteine compared with placebo in patients with IPF. Following the increased mortality in patients with IPF who received IS, CS are considered contraindicated in this specific disease.^14^ Of note, our systematic narrative review includes studies that investigated the utility of BAL and the use of CS in IPF prior to the PANTHER-IPF study.^25 26 37 38 41^ Consequently, these findings from IPF patients should be interpreted with caution, and it is unclear whether they translate to other fILD. Despite the currently limited evidence supporting the diagnostic utility of BAL in IPF and only minimal impact on treatment decision-making, BAL may still provide value in distinguishing IPF from other ILDs, particularly HP.^55^

Our analysis highlights the substantial variability in arbitrarily selected BALL thresholds between 7% and 25% depending on disease context. Although a 15% BALL threshold has been frequently adopted as a conventional reference cut-off, no study to date has prospectively validated this threshold as clinically relevant.^27 43 48^ Moreover, reported BALL thresholds with best prognostic discrimination varied considerably between studies.^28 32^

In general, rather inflammatory ILDs such as HP or sarcoidosis often present with higher BALL counts compared with more fibrotic presentations such as IPF.^54^,^57^ Intermediate BALL values remain diagnostically ambiguous.^56^ Establishing a universal BALL threshold across ILD subtypes is complicated by their heterogeneity and in some cases overlapping pathophysiology. In addition, our findings indicate that CS administration can reduce BALL levels; however, the magnitude of this effect appears to depend on the underlying subtype of ILD and baseline BALL values.^30 34 38 53^ Furthermore, CS and IS treatment at the time of BAL may influence BALL and confound associations with BALL thresholds and treatment responses. However, in most included studies, BAL was performed prior to treatment initiation, suggesting that the overall impact on our findings is likely limited but cannot be fully excluded.

We cannot draw a definitive conclusion, and additional prospective clinical trials are required to address our questions. As a result, the clinical implementation of BALL-based CS treatment decisions remains challenging, given that the current evidence is predominantly derived from retrospective studies and further complicated by the considerable variability in BALL thresholds applied across studies. However, our findings may stimulate prospective clinical studies and, despite current limitations, could still have implications for clinical practice by informing treatment discussions within a multidisciplinary ILD board setting.^30 37 46 51^

Currently, the most reliable approach to predicting CS drug response and determining subsequent outcomes likely involves a combination of different demographic, serological, interventional and radiological features.^1058^,^60^ No specific blood marker has been identified to guide IS treatment or predict long-term outcomes after CS in fILD. Furthermore, the association of BALL and plasma protein expression remains unclear; for example, autoantibodies in SARD-ILD, which indicate systemic disease activity, were not associated with increased BALL levels.^61^

In the BAL, lymphocytes are probably the most trendsetting cell type. Nevertheless, BAL fluid, with its range of cell types, may help elucidate the underlying disease processes and, in some cases, predict drug response.^26 62^ Increased neutrophil counts in the BAL, either alone or in combination with BALL, have been linked to higher rates of disease progression, relapse and mortality.^35 36 39 43 45 54^ In contrast, such associations have not been consistently demonstrated for eosinophils.^35 36 39 45 63^ Elevated eosinophil and neutrophil counts in BAL fluid have been linked to poor therapeutic response to CS treatment.^2846 52^,^54^ But CS can reduce neutrophil and eosinophil counts in BAL in some patients.^49^ However, the correlation between BAL cellularity and histopathological findings of inflammation in fILD remains inconsistent.^25 29 50 60^ During AE-ILD, elevated neutrophil counts in BAL fluid have been associated with a poor prognosis, whereas the presence of BALL is rare but linked to favourable outcomes.^39 48 64^ In clinical practice, neutrophil-predominant BAL profiles may signal limited CS benefit and require careful assessment before starting IS therapy.^3^

However, BAL procedure carries inherent risks, including the potential to trigger AE-ILD, which is associated with poor outcomes and increased mortality.^65^,^67^ Most patients fail to return to their previous clinical, functional and radiological status following AE-ILD.^68^ Therefore, patients undergoing bronchoscopy with BAL should be carefully selected and closely monitored, particularly in the first days to weeks after the procedure.^69^

Imaging modalities, such as high-resolution computer tomography (HRCT), are key components in diagnostic decisions and should be obtained within 6 weeks prior to BAL.^6^ Integrating HRCT findings can help target BAL sampling locations, particularly in radiological heterogeneous ILD presentations.^70^

Radiological imaging with regions showing traction bronchiectasis, reticulations and honeycombing are considered markers of established fibrosis.^70 71^ Ground glass (GG) and consolidations may occasionally reflect inflammatory activity.^10^ Of note, the analysis of the Canadian CARE-PF cohort suggests that BAL cellular distribution poorly correlates with radiological patterns. Thus, challenging a widely held assumption that GG may be a marker of pulmonary inflammation as measured by BAL cellular analysis and suggesting GG may instead represent early fibrotic changes,^6 8^ this retrospective study, focusing on patients with fILD, found no significant association between BALL and radiological features such as GG or reticulations, even when applying a BALL threshold greater than 20%.^8^ Furthermore, BALL can coexist with established fibrosis, suggesting that its presence does not necessarily indicate that predominantly inflammatory mechanisms are the main drivers of disease progression.^8 9^ These two notions underscore the complex and sometimes discordant relationship between BAL cellular distribution, radiological fibrosis and clinical behaviour.

Recent revisions of ILD classification systems have raised new questions regarding the relationship between radiological patterns and alveolar cellularity, particularly in emerging entities, such as bronchiolocentric interstitial pneumonia, which is defined by chronic lymphocyte-predominant peribronchiolar inflammation and may additionally exhibit non-necrotising granulomatous inflammation.^2^ However, its clinical value in the context of specific radiological patterns remains uncertain and warrants further investigation.

Treatment options for fILD remain limited, with IS and antifibrotic therapies commonly used combined if inflammation is suspected and fibrosis has been established.^72^,^74^ However, in different guidelines, CS treatments are currently recommended for patients regardless of their fibrotic component, but the evidence for efficacy and safety in fILD is poor and currently no standardised treatment regimens are recommended or even thoroughly studied.^12 75^ The heterogeneity of clinical practice was striking across the studies we reviewed, with varying CS regimens and frequently incorporated IS combination therapies.^26^,^2834 36 46 49 51^ These different treatment strategies likely have a significant impact on measured outcomes and should be carefully considered when selecting a treatment approach.

### Limitations

Our systematic review is subject to several limitations that warrant careful consideration when interpreting its findings. The main limitations are the quality of the included studies, with the majority exhibiting moderate or high risk of bias, and 60% with a retrospective design, small sample sizes and where BAL are assessed as a secondary outcome, which inherently restricts the robustness and generalisability of the evidence. Additionally, substantial heterogeneity exists among the underlying pulmonary diseases, each characterised by distinct cellular profiles in BAL. This heterogeneity is further compounded by the evolving definitions of these diseases and the changes in their nomenclature over time. Many studies did not clearly define or detail their definition of fibrosis nor specified the exact number of patients with fILD included in their cohorts. Moreover, the retrospective design, varied endpoints and variable treatment regimens of the included studies weaken conclusions about causality between BAL findings and CS response. Additional factors, such as current or past smoking status, cumulative pack-years, patient age and ventilatory status, are known to influence BAL cellular composition.^6376^,^78^ Due to all these reasons, we were not able to perform a meta-analysis and we did not conduct a formal assessment of reporting bias.^18^,^20^

Details regarding the BAL protocols, including fluid volume recovery, instillation site or timing of BAL are largely unavailable in the reviewed studies and may influence cell recruitment and distribution in the lavage.^6 79^ Variability in CS regimens and concurrent IS use across studies further limits the comparability and pooling of clinical outcomes.

Publication bias and selection bias represent another notable limitation, as several potentially relevant studies were not published or could not be included due to unavailability. Moreover, articles published in Korean, Japanese or Mandarin required translation via artificial intelligence, introducing potential inaccuracies or misinterpretations.^37 41 52^

### Implications for research

The available studies are predominantly retrospective and observational, making direct comparisons and interpretation challenging. Well-designed prospective and interventional studies are essential to establish robust evidence for current treatment recommendations. Future research should aim to define standardised BAL protocols, including instillation volume, lobar location and processing techniques, to reduce variability in cellular analysis. Establishing prognostically meaningful BALL thresholds in relation to both diagnosis and treatment response, ideally with an ILD-subtype specific approach, will be critical. Prospective multicentre trials should incorporate BAL cellular profiles as potential stratification criteria or predictive markers in CS or IS trials.

## Conclusions

Current evidence suggests that higher baseline BALL may predict better CS response in fILD, but heterogeneity of ILD subtypes, lack of standardised thresholds and the limited prospective data preclude its use as standalone theragnostic marker. As BAL is a low-risk procedure, further evaluation of BALL as a theragnostic tool is warranted, particularly for integration into multidisciplinary ILD discussions in cases of diagnostic uncertainty or treatment stratification. Standardising thresholds and integrating BAL findings with radiologic and serologic parameters may enable more personalised ILD management.

## Supplementary material

10.1136/bmjresp-2025-004035 online supplemental file 1

10.1136/bmjresp-2025-004035 online supplemental file 2

10.1136/bmjresp-2025-004035 online supplemental file 3

10.1136/bmjresp-2025-004035 online supplemental file 4

10.1136/bmjresp-2025-004035 online supplemental file 5

10.1136/bmjresp-2025-004035 online supplemental file 6

10.1136/bmjresp-2025-004035 online supplemental file 7

## Data Availability

Data are available upon reasonable request.
