# Supplementary material for: BAL lymphocytosis as a predictive marker for drug response and long-term outcome in fibrotic ILD: systematic review
Source: BMJ Open Respir Res. 2026 Jun 4;13(1):e004035. doi: 10.1136/bmjresp-2025-004035 (PMC13239650; doi:10.1136/bmjresp-2025-004035)
Supplement: online supplemental file 1 [file bmjresp-13-1-s001.pdf]

MEDLINE (R) ALL (Ovid)

Ovid MEDLINE(R) ALL <1900 to August 4th, 2025>

1 exp Bronchoalveolar Lavage/ or ((bronchoalveolar or broncho-alveolar or bronchial or pulmonary or lung or alveolar) adj3 (lavage* or washing*)).tw.

2 exp Lung Diseases, Interstitial/ or (acute eosinophilic pneumonia or antisynthetase syndrome or asbestosis or byssinosis or siderosis or anthracosilicosis or berylliosis or coal miner lung or congenital pulmonary lymphangiectasia or letterer-siwe disease or lung sclerosis or lymphangioleiomyomatosis or nonspecific interstitial pneumonia or organizing pneumonia or pleuroparenchymal fibroelastosis or pulmonary alveolar proteinosis or pneumoconios?s or pneumokoniosis or pneumosclerosis or fibrotic hypersensitivity pneumonitis or f-HP or silicos?s or (diffuse adj3 (parenchyma$ or interstitial) adj3 (pulmona* or lung or pneumopath*)) or ((fibrosing or cryptogenic) adj3 (alveoliti* or pneumonia)) or (Pulmonary adj3 histiocytosis) or (idiopathic adj3 fibros?s adj3 (pulmona* or lung or pneumopath*)) or (interstitial adj3 (pulmona* or lung or pneumopath* or pneumoni*)) or (wegener$ adj3 granuloma$) or ((scleroderma or rheumatic$) adj2 (lung$ or pulmonary$ or respiratory$)) or ((pulmonary or lung) adj2 fibros?s)).tw.

3 exp Anti-Inflammatory Agents/ or (steroid* or corticosteroid* or glucocorticoid* or hydroxycorticosteroid* or methylpredniso* or prednisone or prednisolone or betamethasone or fludrocortisone or deflazacort or hydrocortison* or dexamethasone or decadron or dexasone or diodex or hexadrol or fluticasone or budesonide or mometasone or beclomethasone or ciclesonide or ((anti inflammat* or anti-inflammat* or antiinflammat* or antiflogistic or antiphlogistic) adj3 (agent* or drug* or steroid*))).tw.

4 1 and 2 and 3

5 (exp animals/ or exp animal experimentation/ or exp models, animal/ or exp plants/ or exp fungi/) not humans/

6 4 not 5

7 editorial/ or letter/ or editorial.pt. or letter.pt.

8 6 not 7

Embase (El Sevier)

Embase < 1974 to August 4th, 2025>

#8. #6 NOT #7

#7. conference abstract':it OR 'conference paper':it OR 'conference review':it OR 'editorial OR letter':it

#6. #4 NOT #5

#5. animal experiment'/exp OR 'animal model'/exp OR 'experimental animal'/exp OR 'transgenic animal'/exp OR 'male animal'/exp OR 'female animal'/exp OR 'juvenile animal'/exp

#4. #1 AND #2 AND #3

#3. corticosteroid'/exp OR 'antiinflammatory agent'/exp OR 'steroid*':ab,ti OR 'corticosteroid*':ab,ti OR 'glucocorticoid*':ab,ti OR 'hydroxycorticosteroid*':ab,ti OR 'methylpredniso*':ab,ti OR 'prednisone':ab,ti OR 'prednisolone':ab,ti OR 'betamethasone':ab,ti OR 'fludrocortisone':ab,ti OR 'deflazacort':ab,ti OR 'hydrocortison*':ab,ti OR 'dexamethasone':ab,ti OR 'decadron':ab,ti OR 'dexasone':ab,ti OR 'diodex':ab,ti OR 'hexadrol':ab,ti OR 'fluticasone':ab,ti OR 'budesonide':ab,ti OR 'mometasone':ab,ti OR 'beclomethasone':ab,ti OR 'ciclesonide':ab,ti OR ((('anti inflammat*' OR 'anti inflammat*' OR antiinflammat* OR antiflogistic OR antiphlogistic) NEAR/3 (agent* OR drug* OR steroid*)):ab,ti)

#2. "interstitial lung disease'/exp OR 'interstitial pneumonia'/exp OR 'fibrosing alveolitis'/exp OR (('acute eosinophilic pneumonia':ab,ti OR 'antisynthetase syndrome':ab,ti OR 'asbestosis':ab,ti OR 'byssinosis':ab,ti OR 'siderosis':ab,ti OR 'anthracosilicosis':ab,ti OR 'berylliosis':ab,ti OR 'coal miner lung':ab,ti OR 'congenital pulmonary lymphangiectasia':ab,ti OR 'letterer-siwe disease':ab,ti OR 'lung sclerosis':ab,ti OR 'lymphangioleiomyomatosis':ab,ti OR 'nonspecific interstitial pneumonia':ab,ti OR 'organizing pneumonia':ab,ti OR 'pleuroparenchymal fibroelastosis':ab,ti OR 'pulmonary alveolar proteinosis':ab,ti OR 'pneumoconios?s':ab,ti OR 'pneumokoniosis':ab,ti OR 'pneumosclerosis':ab,ti OR 'fibrotic hypersensitivity pneumonitis':ab,ti OR 'f-hp':ab,ti OR 'silicos?s':ab,ti) AND ((diffuse NEAR/3 (parenchyma$ OR interstitial) NEAR/3 (pulmona* OR lung OR pneumopath*)):ab,ti)) OR (((fibrosing OR cryptogenic) NEAR/3 (alveoliti* OR pneumonia)):ab,ti) OR ((pulmonary NEAR/3 histiocytosis):ab,ti) OR ((idiopathic NEAR/3 fibros?s NEAR/3 (pulmona* OR lung OR pneumopath*)):ab,ti) OR ((interstitial NEAR/3 (pulmona* OR lung OR pneumopath* OR pneumoni*)):ab,ti) OR ((wegener$ NEAR/3 granuloma$):ab,ti) OR (((scleroderma OR rheumatic$) NEAR/2 (lung$ OR pulmonary$ OR respiratory$)):ab,ti) OR (((pulmonary OR lung) NEAR/2 fibros?s):ab,ti)"

#1. bronchoalveolar lavage fluid'/de OR (((bronchoalveolar OR 'broncho alveolar' OR bronchial OR pulmonary OR lung OR alveolar) NEAR/3 (lavage* OR washing*)):ab,ti)

Web of Science (Clarivate)

Web of science < 1900 to August 4th, 2025>

1 "TS=(Anti-inflammatory OR Anti nflammatory OR Antiinflammatory)"

2 "TI=(Anti-inflammatory OR Anti inflammatory OR Antiinflammatory OR steroid* OR corticosteroid* OR glucocorticoid* OR hydroxycorticosteroid* OR methylpredniso* OR prednisone OR prednisolone OR betamethasone OR fludrocortisone OR deflazacort OR hydrocortison* OR dexamethasone OR decadron OR dexasone OR diodex OR hexadrol OR fluticasone OR budesonide OR mometasone OR beclomethasone OR ciclesonide)"

3 "AB=(Anti-inflammatory OR Anti inflammatory OR Antiinflammatory OR steroid* OR corticosteroid* OR glucocorticoid* OR hydroxycorticosteroid* OR methylpredniso* OR prednisone OR prednisolone OR betamethasone OR fludrocortisone OR deflazacort OR hydrocortison* OR dexamethasone OR decadron OR dexasone OR diodex OR hexadrol OR fluticasone OR budesonide OR mometasone OR beclomethasone OR ciclesonide)"

4 "#1 OR #2 OR #3"

5 "AB=(histiocytosis OR pneumoconiosis OR asbestosis OR byssinosis OR siderosis OR anthracosilicosis OR berylliosis OR lymphangiectasia OR pneumoconios?s OR pneumokoniosis OR pneumosclerosis OR silicos?s)"

6 "TI=(histiocytosis OR pneumoconiosis OR asbestosis OR byssinosis OR siderosis OR anthracosilicosis OR berylliosis OR lymphangiectasia OR pneumoconios?s OR pneumokoniosis OR pneumosclerosis OR silicos?s)"

7 "AB=((pulmonary OR lung) NEAR/2 (fibros?s))"

8 "TI=((pulmonary OR lung) NEAR/2 (fibros?s))"

9 "AB=((interstitial) NEAR/2 (pulmona* OR lung OR pneumopath* OR pneumoni*))"

10 "TI=((interstitial) NEAR/2 (pulmona* OR lung OR pneumopath* OR pneumoni*))"

11 "TS=(interstitial lung disease OR ((pulmonary OR lung) NEAR/2 (fibros?s)))"

12 "#5 OR #6 OR #7 OR #8 OR #9 OR #10 OR #11"

13 "AB=((bronchoalveolar OR bronchial OR pulmonary OR lung OR alveolar) NEAR/2 (lavage* OR washing*))"

14 "TI=((bronchoalveolar OR bronchial OR pulmonary OR lung OR alveolar) NEAR/2 (lavage* OR washing*))"

15 "TS=(bronchoalveolar lavage fluid)"

16 "#13 OR #14 OR #15"

17 "#4 AND #12 AND #16"

Google scholar

< August 4th, 2025>

Not including citations, sorted by relevance, the first 200 articles were exported directly from Google Scholar

bronchoalveolar lavage fluid|pulmonary lavage AND interstitial lung disease|pulmonary fibrosis|histiocytosis|pneumoconiosis|asbestosis|byssinosis|siderosis|anthracosilicosis|pneumoconiosis|pneumosclerosis|silicosis AND corticosteroid|antiinflammatory
