# Supplementary material for: BAL lymphocytosis as a predictive marker for drug response and long-term outcome in fibrotic ILD: systematic review
Source: BMJ Open Respir Res. 2026 Jun 4;13(1):e004035. doi: 10.1136/bmjresp-2025-004035 (PMC13239650; doi:10.1136/bmjresp-2025-004035)
Supplement: online supplemental file 3 [file bmjresp-13-1-s003.docx]

**Supplement 3**

**Inclusion and exclusion criteria**

The eligibility assessment followed pre-defined inclusion criteria which required studies to include patients with fILD who had undergone bronchoscopy with BAL and who were receiving CS either alone or in combination with other immunosuppressive therapy (IS) agents. Studies lacking CS treatment or BAL procedure were excluded. Studies conducted exclusively in ILD patients without any fibrotic component were excluded, as were studies with insufficient or non-extractable data regarding BALL or CS treatment outcomes. Editorials and commentaries were excluded from consideration. Eligibility for inclusion in the review was limited to clinical trials and observational studies.

The systematic review was conducted in accordance with the procedures outlines in the study protocol and no protocol changes were made after registration.

**Data extraction**

A data extraction form was designed in Excel sharepoint to retrieve the following information: author, year of publication, journal, language, and country of origin, study design, duration of follow-up. Additionally, clinical details were recorded, such as the underlying fILD, sample sizes, type of intervention, and the reported endpoints. A summary of the main findings from each study was also registered. The findings were classified into three categories: BALL as a predictor of CS response, the determination and variability of BALL thresholds across studies, and the effect of CS treatment on BALL levels. The measurable effects of interest were extracted, if possible, with the corresponding 95% confidence intervals.
