# Supplementary material for: BAL lymphocytosis as a predictive marker for drug response and long-term outcome in fibrotic ILD: systematic review
Source: BMJ Open Respir Res. 2026 Jun 4;13(1):e004035. doi: 10.1136/bmjresp-2025-004035 (PMC13239650; doi:10.1136/bmjresp-2025-004035)
Supplement: online supplemental file 5 [file bmjresp-13-1-s005.docx]

Supplement 5: detailed description of included studies

| **Author** | **Study type** | **Disease** | **Number of patients (n) / number of patient with BAL** | **Smoking status (n=active/ former/ never)** | **IS/CS before BAL or intervention (n)** | **CS (regime if known) / other IS** | **Follow-up time** | **Risk of bias** |
| --- | --- | --- | --- | --- | --- | --- | --- | --- |
| Bacha *et al*^30^ | prospective ChS | Sarcoidosis | 40/40 | 13/0/27 | No | NR / NR | 12±9 months | High (S3 C0 O2) |
| Behr *et al*^31^ | prospective ChS | SSc | 79/79 | 3/4/72 | No (72), not for at least 3 months before IS (7) | Prednisone (1mg/kg/d for 8 to 12 weeks) / NR | 56.8±3.1 weeks | High (S3 C1 O2) |
| Cho *et al*^27^ | retrospective ChS | NSIP | 204/159 | 18/29/157 | NR | NR / azathioprine, cyclophosphamide | 70.9±10.8 months | High (S2 C0 O2) |
| Cho *et al*^52^ | retrospective ChS | IPF/SARD-ILD | 31/31 | 14/0/17 | No | NR / NR | 3-18 months | High (S2 C0 O2) |
| De Sadeleer *et al*^32^ | retrospective ChS | fHP | 91/91 | NR | No | NR / NR | Lung function at 12 months or treatment interruption, survival 130 months | Moderate (S3 C1 O3) |
| Drent *et al*^33^ | retrospective ChS | Sarcoidosis | 26/26 | 0/0/26 | No | NR / NR | 9 to 34 months | High (S2 C0 O3) |
| Giacomelli *et al*^34^ | prospective ChS | SSc | 23/23 | 0/0/23 | No | Prednisone (25mg/d for 1 month, followed bz 5 mg/d for 5 months) / cyclophosphamide | 6 months | High (S2 C0 O2) |
| Goh *et al*^35^ | retrospective ChS | SSc | 141/141 | 0/45/96 | Yes 36, No 105 | Prednisone (>1mg/d) / azathioprine, mycophenolate mofetil, cyclophosphamide | 10 years | High (S2 C0 O2) |
| Greene *et al*^36^ | prospective ChS | SARD-ILD | 36/36 | 12/24/0 | No | Prednisone (40 mg/d) / cyclophosphamide | Lung function 2 months, clinical evaluation 18 months | High (S3 C0 O2) |
| Haslam *et al*^26^ | retrospective ChS | IPF, SARD-ILD | 66 (36 IPF, 15 SARD-ILD) / 66 | 24/12/15 | Yes 10, No 26, NR 30 | Prednisone / NR | 12 months | Moderate (S3 C1 O3) |
| Haslam *et al*^25^ | prospective ChS | IPF, asbestosis | 21 (18 IPF, 3 asbestosis) / 21 | 7/8/6 | Yes 3, No 18 | Prednisone (40-60mg/d for minimum 4 weeks) / NR | 12 months | Moderate (S3 C0 O3) |
| He *et al*^29^ | retrospective ChS | Dermatomyositis-ILD | 113 (27 AIP, 86 CIP) / 113 | 18/0/95 | Yes 83, No 30 | Methylprednisolone (500mg/d for 3 days) / NR | 22±14.9 months | Moderate (S3 C0 O3) |
| Kang *et al*^37^ | prospective ChS | IPF | 20 / 20 | 20/0/0 | NR | Prednisone (1mg/kg/d for 8-12 weeks followed by 0.25mg/kg/d) / cyclophosphamide | 6 months | Moderate (S3 C0 O3) |
| Karpel *et al*^38^ | prospective ChS | IPF | 18 (10 IPF, 8 healthy patients) / 18 | 0/3/15 | No | Prednisone (1mg/kg/d) / NR | 2±0.5 months | Moderate (S4 C1 O2) |
| Kase *et al*^53^ | retrospective Chs | SSc | 68 / 68 | 3/22/43 | Yes 20, No 48 | Prednisone / cyclophosphamide, methotrexate | 88±30 months | Moderate (S3 C1 O3) |
| Kono *et al*^39^ | retrospective ChS | AE-ILD | 71 (26 AE-IPF, 45 AE-non-IPF) / 71 / 22 | 5/44/21 | Yes 10, No 61 | Methylprednisolone 1000mg/d for 3 days) / NR | 3 and 12 months | Moderate (S3 C0 O3) |
| Kurasawa *et al*^40^ | prospective ChS | Dermatomyositis and polymyositis-ILD | 22 with IP (13 DM, 9 PM), 12 without IP (4 DM, 8 lung cancer) | NR | No | Prednisone 1mg/kg/d for 4 weeks, followed by tapering) / NR | 12-48 months | High (S2 C0 O2) |
| Kyung *et al*^41^ | retrospective ChS | IPF | 36 / 36 | 9/12/11^a^ | NR | NR / NR | 3 years | Moderate (S3 C1 O3) |
| Lewandowska *et al*^28^ | retrospective ChS | HP | 93 (54 fHP, 39 non-fHP) / 78 | 78^b^/0/15 | NR | Prednisone (0.5mg/kg/d, gradually tapered) / azathioprine | 6-12 and 200 months | Moderate (S3 C1 O2) |
| Li *et al*^42^ | retrospective ChS | IPF | 126 / 126 | 75/0/51 | NR | NR / NR | 29.6 months | Low (S3 C2 O3) |
| Matsuo *et al*^43^ | retrospective ChS | IIP | 35 / 29 | NR | No | Prednisone (1-1.5mg/kg/d for 1 month, tapered thereafter at 5 mg per month / NR | NR | High (S2 C0 O2) |
| Newman *et al*^44^ | prospective ChS | Beryllium disease | 110 (Beryllium disease 55, beryllium-sensitized without disease 8, control 47) / 110 | 48/39/23 | Yes 10, No 100 | Prednisone (5-60mg/d) / NR | NR | High (S2 C0 O2) |
| Novoa-Bolivar *et al*^54^ | retrospective Chs | HP, sarcoidosis, COP, LIP, RB-ILD, DIP, NSIP, pneumoconiosis, PLCH, eosinophilic ILD, unclassifiable ILD, AIP | 1074 / 371 | NR | NR | NR / rituximab, azathioprine, mycophenolate mofetil, cyclophosphamide, tacrolimus | NR | Moderate (S3 C1 O3) |
| Onishi *et al*^45^ | retrospective ChS | OP | 75 / 75 | 6/32/36^a^ | Yes 75 | Prednisone (0-5-0.8 mg/kg/d, tapered to 5-10mg/d within 1-2 weeks, total administration 1-6 months) / NR | NR | Moderate (S3 C0 O3) |
| Rudd *et al*^46^ | prospective ChS | IPF | 120 / 36 | NR | No | Prednisone (40-60mg/d) / cyclophosphamide, azathioprine, penicillamine | 38 months | Moderate (S2 C0 O2) |
| Sharma *et al*^47^ | retrospective ChS | Chronic silicosis | 51 / 38 | 18/0/33 | No | Prednisone (30mg/d, followed by tapering of 2.5 mg/week until 0.25mg/kg/d) / NR | 1.5, 3, 6 months | High (S2 C0 O2) |
| Takei *et al*^48^ | retrospective ChS | AE chronic fILD | 37 / 28 | 4/23/9^a^ | Yes 6, No 31 | Methylprednisolone (1000mg/d for 3 days) / cyclophosphamide, cyclosporine | 6.9±4.4 months | High (S3 C0 O2) |
| Turner-Warwick *et al*^49^ | prospective ChS | IPF and SARD-ILD | 32 (26 IPF, 6 SARD-ILD) / 32 | 26/0/6 | Yes 6, No 26 | Prednisone (20-60mg for 4 weeks, followed by tapering over 12 weeks to minimal 20 mg alternate day) / cyclophosphamide | 4±1.5years | High (S2 C1 O2) |
| Watters *et al*^50^ | prospective ChS | IPF | 26 / 26 | 12/0/14 | No | Prednisone (1.5mg/kg/d for 6 weeks, followed by 1mg/kg/d for 6 weeks, followed by 0.5mg/kg/d for 3 months, gradual tapering to 0.25mg/kg/d) / NR | 6 and 12 months | Moderate (S3 C1 O2) |
| Yamagata *et al*^51^ | retrospective ChS | NSIP, PPFE and unclassifiable IIP | 186 (37 NSIP, 16 PPFE, 133 unclassifiable IIP) / 186 | 100/0/86 | No | NR / NR | 4.4±2.3 years | Moderate (S3 C1 O3) |
