# Supplementary material for: BAL lymphocytosis as a predictive marker for drug response and long-term outcome in fibrotic ILD: systematic review
Source: BMJ Open Respir Res. 2026 Jun 4;13(1):e004035. doi: 10.1136/bmjresp-2025-004035 (PMC13239650; doi:10.1136/bmjresp-2025-004035)
Supplement: online supplemental file 6 [file bmjresp-13-1-s006.docx]

Supplement 6: Reported Thresholds for BAL Lymphocytosis

| **Author** | **Study type** | **Disease** | **Number of patients (N)** | **BALL threshold before analysis** | **Calculated BALL threshold** |
| --- | --- | --- | --- | --- | --- |
| Behr *et al.* | Prospective ChS | SSc | 79 | >15% |  |
| Cho *et al.* | Retrospective ChS | fNSIP | 204 | >15% |  |
| De Sadeleer *et al.* | Retrospective ChS | fHP | 91 | >20% | >20% |
| Goh *et al.* | Retrospective ChS | SSc | 141 | >14% |  |
| Haslam *et al.* | Retrospective ChS | IPF, SARD-ILD | 66 | >20% | >11% |
| Haslam *et al.* | Prospective ChS | IPF, asbestosis | 21 |  | >5% |
| Kono *et al.* | Retrospective ChS | AE-ILD | 71 |  | >25% |
| Kurasawa *et al.* | Prospective ChS | DM-ILD, PM-ILD | 34 | >15% |  |
| Lewandowska *et al.* | Retrospective ChS | HP | 93 |  | >54% |
| Matsuo *et al.* | Retrospective ChS | IIP | 35 | >15% |  |
| Novoa-Bolivat *et al.* | Retrospective Chs | HP, sarcoidosis, COP, LIP, RB-ILD, DIP, fNSIP, unclassificalbe ILD | 1074 |  | >7% |
| Rudd *et al.* | Prospective ChS | IPF | 120 | >11% |  |
| Takei *et al.* | Retrospective ChS | AE-ILD | 37 | >15% |  |
| Turner-Warwick *et al.* | Prospective ChS | IPF, SARD-ILD | 32 | >11% |  |
| Watters *et al.* | Prospective ChS | IPF | 26 | >13% |  |
| Yamagata *et al.* | Retrospective ChS | iNSIP, iPPFE, unclassifiable IIP | 186 | >15% | >16.6% |

AE-ILD: acute exacerbation of interstitial lung disease, BALL: bronchoalveolar lavage lymphocytosis, ChS: cohort study, COP: cryptogenic organizing pneumonia, DIP: desquamative interstitial pneumonia, DM: dermatomyositis, fHP: fibrotic hypersensitivity pneumonia, fNSIP: fibrotic non-specific interstitial pneumonia, ILD: interstitial lung disease, IP: interstitial pneumonia, IPF: idiopathic pulmonary fibrosis, LIP: lymphocytic interstitial pneumonia, PM: polymyositis, RB-ILD: respiratory bronchiolitis interstitial lung disease, SARD-ILD: Systemic Autoimmune Rheumatic Diseases-associated Interstitial Lung Disease, SSc: systemic sclerosis
