# Supplementary material for: BAL lymphocytosis as a predictive marker for drug response and long-term outcome in fibrotic ILD: systematic review
Source: BMJ Open Respir Res. 2026 Jun 4;13(1):e004035. doi: 10.1136/bmjresp-2025-004035 (PMC13239650; doi:10.1136/bmjresp-2025-004035)
Supplement: online supplemental file 7 [file bmjresp-13-1-s007.docx]

Supplement 7: Effect of Corticosteroid Therapy on BALL Levels

| **Author** | **Intervention (N)** | **Risk of bias assessment** | **BALL before – after IS** |
| --- | --- | --- | --- |
| Bacha *et al*^30^ | CS (40) | High | 67% ± 19.2 vs 40.75% ± 17.45 (p<0.05) |
| Giacomelli *et al*^34^ | CS and cyclophosphamide (23) | High | CS 16% (6-77%) vs 12 % (5-80%) (NS) |
| Karpel *et al*^38^ | CS (10) | Moderate | Significant decrease of BALL after CS |
| Sharma *et al*^47^ | CS (34) | High | 25.86 ±16.5 vs 20.29 ± 15.67 (NS)  Significant decrease in BALL in patients with complicated silicosis (p<0.05). |
| **Author** | **Intervention (N)** |  | **BALL between untreated vs treated patients** |
| He *et al*^29^ | CS (23) | Moderate | 29.5% (8.5 – 51.5) vs 12.8% (6.8— 34.8), p<0.05) |
| Kase *et al*^53^ | CS (18), CS and cyclosporine (1), methotrexate alone (1) | Moderate | 11.9% vs 10%, NS. |
| Newman *et al*^44^ | CS (10) | High | 46% ± 29% vs 41% ± 22% (NS) |

BALL: bronchoalveolar lavage lymphocytosis, CS: corticosteroids, DLCO: Diffusing Capacity of the Lungs for Carbon Monoxide, N: number of patients, NS: non-significative
